# Supplementary material for: Bayesian refinement of protein structures and ensembles against SAXS data using molecular dynamics
Source: PLoS Comput Biol. 2017 Oct 18;13(10):e1005800. doi: 10.1371/journal.pcbi.1005800 (PMC5662244; doi:10.1371/journal.pcbi.1005800)
Supplement: S1 Archive — (BZ2) [file pcbi.1005800.s011.tar.bz2 › gromacs_4.62_bayesian_saxs_refi/share/html/online/g_bond.html]

g\_bond


| g\_bond Main Table of Contents |

**VERSION 4.6  
Sat 19 Jan 2013**

---

### Description

g\_bond makes a distribution of bond lengths. If all is well a
Gaussian distribution should be made when using a harmonic potential.
Bonds are read from a single group in the index file in order i1-j1
i2-j2 through in-jn.

-tol gives the half-width of the distribution as a fraction
of the bondlength (-blen). That means, for a bond of 0.2
a tol of 0.1 gives a distribution from 0.18 to 0.22.

Option -d plots all the distances as a function of time.
This requires a structure file for the atom and residue names in
the output. If however the option -averdist is given (as well
or separately) the average bond length is plotted instead.

### Files

| option | filename | type | description |
| --- | --- | --- | --- |
| **-f** | traj.xtc | Input | Trajectory: xtc trr trj gro g96 pdb cpt |
| **-n** | index.ndx | Input | Index file |
| **-s** | topol.tpr | Input, Opt. | Structure+mass(db): tpr tpb tpa gro g96 pdb |
| **-o** | bonds.xvg | Output | xvgr/xmgr file |
| **-l** | bonds.log | Output, Opt. | Log file |
| **-d** | distance.xvg | Output, Opt. | xvgr/xmgr file |

### Other options

| option | type | default | description |
| --- | --- | --- | --- |
| **-[no]h** | bool | no | Print help info and quit || **-[no]version** | bool | no | Print version info and quit || **-nice** | int | 19 | Set the nicelevel || **-b** | time | 0 | First frame (ps) to read from trajectory || **-e** | time | 0 | Last frame (ps) to read from trajectory || **-dt** | time | 0 | Only use frame when t MOD dt = first time (ps) || **-[no]w** | bool | no | View output .xvg, .xpm, .eps and .pdb files || **-xvg** | enum | xmgrace | xvg plot formatting: xmgrace, xmgr or none || **-blen** | real | -1 | Bond length. By default length of first bond || **-tol** | real | 0.1 | Half width of distribution as fraction of -blen || **-[no]aver** | bool | yes | Average bond length distributions || **-[no]averdist** | bool | yes | Average distances (turns on -d) |

### Known problems

- It should be possible to get bond information from the topology.

---

http://www.gromacs.org  
gromacs@gromacs.org
